# Zebrafish: a vertebrate tool for studying basal body biogenesis, structure, and function

**DOI:** 10.1186/s13630-016-0036-2

**Published:** 2016-05-10

**Authors:** Ryan A. Marshall, Daniel P. S. Osborn

**Affiliations:** Cell Sciences and Genetics Research Centre, St George’s University of London, London, SW17 0RE UK

**Keywords:** Zebrafish, Basal bodies, Crispr, Centriole duplication, Ultrastructure, Ciliopathies

## Abstract

Understanding the role of basal bodies (BBs) during development and disease has been largely overshadowed by research into the function of the cilium. Although these two organelles are closely associated, they have specific roles to complete for successful cellular development. Appropriate development and function of the BB are fundamental for cilia function. Indeed, there are a growing number of human genetic diseases affecting ciliary development, known collectively as the ciliopathies. Accumulating evidence suggests that BBs establish cell polarity, direct ciliogenesis, and provide docking sites for proteins required within the ciliary axoneme. Major contributions to our knowledge of BB structure and function have been provided by studies in flagellated or ciliated unicellular eukaryotic organisms, specifically *Tetrahymena* and *Chlamydomonas.* Reproducing these and other findings in vertebrates has required animal in vivo models. Zebrafish have fast become one of the primary organisms of choice for modeling vertebrate functional genetics. Rapid ex-utero development, proficient egg laying, ease of genetic manipulation, and affordability make zebrafish an attractive vertebrate research tool. Furthermore, zebrafish share over 80 % of disease causing genes with humans. In this article, we discuss the merits of using zebrafish to study BB functional genetics, review current knowledge of zebrafish BB ultrastructure and mechanisms of function, and consider the outlook for future zebrafish-based BB studies.

## Body of the primer

### *Zebrafish (Danio rerio):* what is the basic phylogeny of this organism?

The zebrafish has been employed to study not only vertebrate development, genetics, and disease but, due to the comprehensive genomic annotation, has also helped answer questions of evolutionary diversity and phylogeny [1]. In short, zebrafish (*Danio rerio*), exhibit a toothless jaw that classifies them under the Cyprinidae family, with other members including carp, barbs, and minnows [2]. The Cyprinids themselves fall under the order of *Cypriniformes*, a large and diverse grouping of ray-finned (class: *Actinopterygii***)** bony freshwater fishes [3]. The presence of a swim bladder for buoyancy, moveable jaw, and symmetrical caudal fin classifies zebrafish under the subdivision (or infraclass) of *Teleostei.* There are currently approximately 26,840 species of Teleosts that represent 96 % of all living fish species spread across 40 orders, 448 families, and 4278 genera [4]. The successful evolutionary advance of Teleost fishes has been attributed to the occurrence of a whole genome duplication (WGD) that appeared early in the evolution of ray-finned fish, during the divergence from the lobe-finned fish, some 320–400 million years ago [5, 6]. It is generally accepted that WGD created new evolutionary opportunity by increasing gene number without affecting gene dosage [6]. Consequently, WGD allowed for the introduction of new loci with potentially advantageous functions, accounting for genetic redundancy. Whilst WGD created an expansion of genetic material and permitted leaps in evolutionary advancement, it has complicated analyses of gene function and phylogeny, especially in the context of human disease. Indeed, zebrafish possess at least one orthologue of approximately 70 % of all human genes (roughly 40 % of which have been duplicated) and 82 % of human disease causing genes [7]. However, idiosyncrasies taken into account, zebrafish offer a tractable system for studying gene function as indicated by the clear expansion in zebrafish functional genetics, notably in recent years, into the field of cilia and BB biology.

### Basic basal body structure

**Fig. 1:**
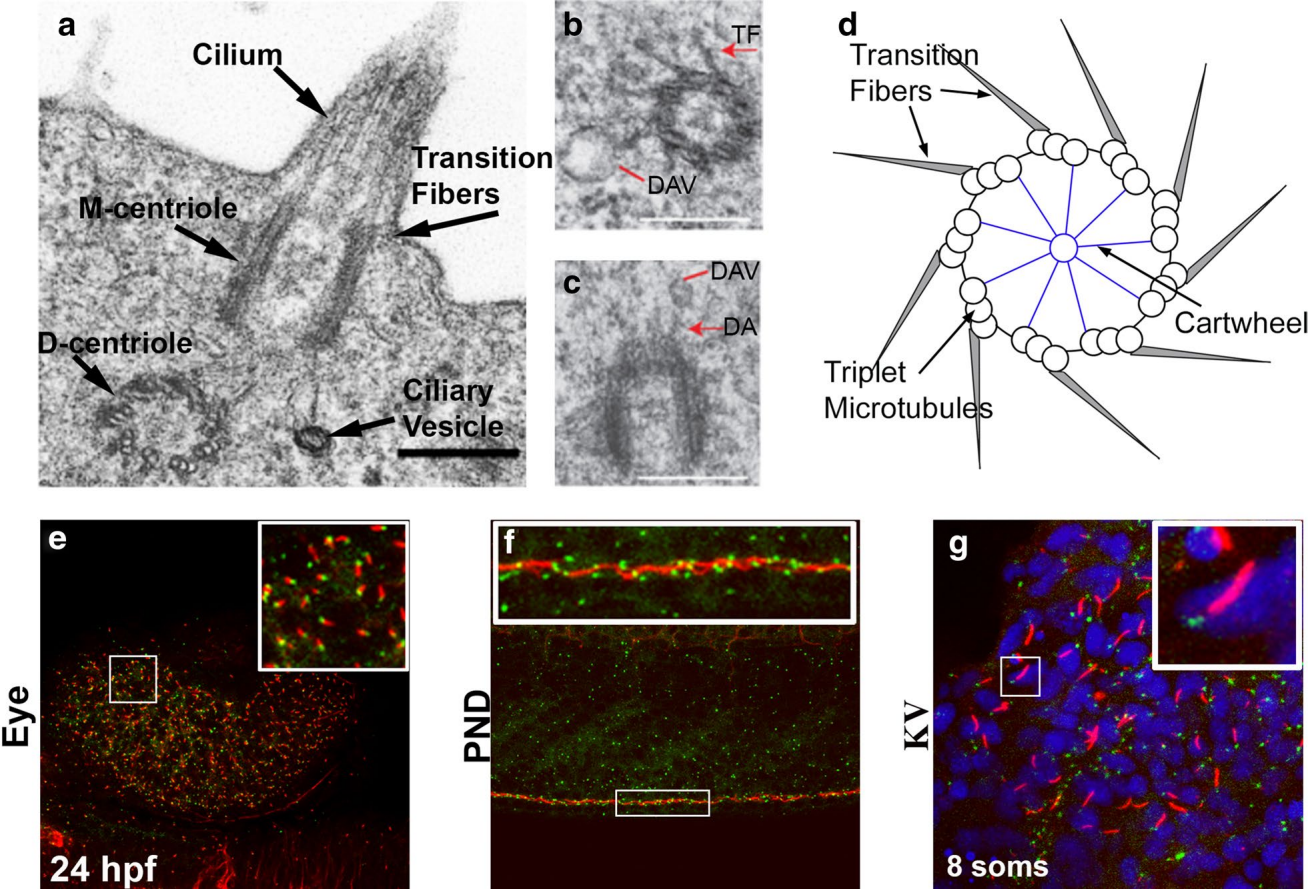
**a**–**d** Zebrafish transmission electron micrographs highlighting conserved BB structures: nine-triplet microtubule arrangement, *TF* transition fibers, *DA* distal appendages, *DAV* distal appendage vesicles. **a** Ultrastructure of the BBs and cilium from the zebrafish brain at 24 hpf. *Scale bar* 250 nm. **b**, **c** M-centrioles from zebrafish photoreceptors at 50 hpf. *Scale bar* 250 nm. **d** Schematic representation of zebrafish BB ultrastructure. **e**–**g** BBs and cilia can be simultaneously visualized in multiple zebrafish tissue types using GTU88 γ**-**Tubulin (BB) and acetylated α-Tubulin (cilia) antibodies. Fluorescent immunohistochemistry in the eye (**e**), pronephric duct (**f**), Kupffer’s vesicle (**g**) for BB (*green*), cilia (*red*) and nuclei (*blue*) in 24 hpf (**e**, **f**) 8 somite (**g**) embryos Reprinted from [15]. Reprinted from [16]

Consisting of a barrel-shaped centriole tethered to the cell membrane, the BB is fundamental in directing ciliogenesis, cell polarity, and providing a docking site for essential intraflagellar transport (IFT) proteins, required for appropriate ciliary function [8–10]. The centriole structure is highly conserved across species and is composed of nine triplet microtubules arranged in a cylindrical shape [11]. This structure forms the template that nucleates the ciliary axoneme. Therefore, correct BB construction dictates the development and function of the cilium. Much of the pioneering work on BB ultrastructure comes from detailed transmission electron microscopy (TEM) from the unicellular flagellate *Chlamydomonas* and the ciliated protozoa *Tetrahymena* [12, 13]. There is, however, very little high-resolution data on the ultrastructure of the BB in zebrafish and vertebrates as a whole. The majority of zebrafish TEM studies in the field of ciliogenesis have focused on axonemal structure of the cilium, which conforms to the nine plus two and nine plus zero doublets associated with motile and primary cilia, respectively [14]. Therefore, it might be speculated that BB structure also conforms to the nine triplet microtubular arrangement. Indeed, this is what is observed in BBs from modified primary cilia in the eye and motile cilia located in the choroid plexus, required for cerebrospinal fluid movement, in the brain (Fig. 1a–d) [15, 16]. Further conservation of structural function has been suggested from closer inspection of the cartwheel architecture, which forms the scaffold at the center of the BB. Sas-6, which localizes to the cartwheel that is required for early BB biogenesis in multiple model systems [17–20]. Interestingly, zebrafish Sas-6 protein has been observed to self-assemble in vitro into structures reminiscent of the cartwheel structure, suggesting Sas-6 itself is a major contributor to the core structural organization at the center of zebrafish BBs [21]. However, despite some compelling BB findings in zebrafish, further studies focusing on BB ultrastructure need to be conducted to elucidate BB structure variants between organisms and within different tissue types.

### Additional basal body structures or accessory structures

Electron microscopy has been fundamental to BB discovery. Descriptive TEM observations of *Tetrahymena* BBs nearly 50 years ago identified structural off-shoots that were speculated to be required for BB orientation and function [13]. These structures include the rootlet, basal foot, postciliary microtubules, transition fibers, and kinetodesmal fibers. Whilst the functional roles of these accessory structures remain largely unknown, there is growing evidence that they play a role in BB orientation, microtubular organization, ciliary structural support, and anchoring [22–24]. Some of these structures have been identified in zebrafish TEM, such as the rootlet, distal appendages, transition fibers, and basal foot (Fig. 1a–d) [16, 25]. However, the zebrafish model has yet to be exploited to specifically focus on accessory structure morphology and function.

### Basal body origins and life cycle

BBs are closely related to centrosomes, they are structurally similar and both act as microtubule organizing centers. In fact, they are largely considered the same entity that has simply taken on a different cellular role post-mitotically, representing an efficient use of cellular components. It has yet to be determined when exactly in zebrafish development BBs become established. However, cilia are first observed during late epiboly, at the initiation of convergence and extension when cellular movements form the embryonic germ layers [26]. Despite this, it is well documented that the reassignment of occupation, from perinuclear centrosomal function to the apical membrane for ciliogenesis, occurs across species. Distinct cellular cues are likely to co-ordinate this event; however, the mechanism of centriole migration and BB docking to the apical membrane is not fully understood. Several studies in zebrafish have helped to identify some novel players in this process, including the Rac1 nucleotide exchange complex ELMO–DOCK1, and the Hippo pathway [27, 28]. Functional knockdown of *elmo1*, *dock1*, or *ezrin1* (components of the ELMO–DOCK1 complex), using antisense morpholino oligonucleotides, results in morphological defects consistent with cilia loss [27]. Morphant embryos display detached BBs at the apical membrane and impaired ciliary axoneme formation. Similarly, the Hippo pathway transcriptional co-activator *yes*-*associated protein(yap)* has been shown to be required for appropriate BB arrangement and apical membrane docking during zebrafish ciliogenesis [29]. Examination of the cross-talk and interactions between the proteins proposed to orchestrate correct BB migration and docking will help clarify this poorly understood process.

Duplication of BBs occurs during mitosis. In multiciliated cells (MCCs), BB number directly underpins the sum of motile cilia, thus proposing the quandary; how do multiple BBs form without cell division? Deuterosomes, electron-dense structures, are believed to drive centriole amplification in MCCs [30]. Deuterosomes have yet to be directly observed in zebrafish and it may be speculated that an alternative method for centriole amplification is employed here. Indeed, *Deuterosome protein 1(Deup1),* required for deuterosome-dependent (DD) centriole biogenesis, is not present in zebrafish [31]. Interestingly, *cep63* required for mother centriole duplication (MCD) is present in zebrafish [31]. *Deup1* and *cep63* are known to be paralogues with divergent roles in MCC promotion. The presence of *cep63* but not *deup1* in zebrafish suggests that *Deup1* arose from *cep63* and that zebrafish amplify their centrioles via MCD, this is likely since zebrafish MCC only contain a few cilia [31]. However, what environmental cues instruct a cell to start amplifying centrioles? Cells are singled out to become MCC through inhibition of notch/delta signaling. Notch regulates Multicilin that promotes the production of centriolar structural proteins and *foxj1*, required for basal body docking, cilia formation and motility [32, 33]. In zebrafish, the *foxj1a* (the homologue of the mammalian *Foxj1*) target *geminin coiled*-*coil domain containing (gmnc)* has been identified to be required for MCC formation [32]. Fish with disrupted *gmnc* fail to generate MCC, lack cells containing multiple BBs and develop cystic kidneys, due to the requirement for MCCs to propel filtrate along the zebrafish pronephric tubule [32]. This suggests that *gmnc* is a critical regulator of centriole amplification. Thus, a cascade of gene regulation is required to promote centriole amplification and ultimately MCC commitment. However, the regulated decision to activate this cascade, independent of cell division, remains unclear.

### Identification of basal body components

Determining the structural protein composition of BBs has often been a complex task, mainly due to difficulties isolating matrix-embedded centrioles from surrounding contaminants for proteomic analysis. In particular, proteins that make up the amorphic pericentriolar material (PCM) can often obscure centriole-specific proteins [34], However, some clever approaches have been used to piece together the ingredients that make up the BB. Several studies have taken a comparative genomics approach to identify genome differences between ciliated and non-ciliated species [35, 36]. Whilst this predicts the required ciliary components, it does not dissect out BB-specific centriolar proteins. A much more direct approach has been used in *Tetrahymena* and *Chlamydomonas*, where minimal PCM has aided BB isolation allowing mass spectroscopy to identify more specific BB proteome candidates [34, 37]. This has been highly informative in identifying a “parts list” for basal body assembly. Whilst similar experiments have not been conducted in zebrafish, high conservation in centriole function and therefore protein content should permit vertebrate follow-up experiments. In recent years, the multinational consortium known as SYSCILIA has compiled a “Gold standard” (SCGS) list of ciliary components found in the human genome [38]. For this article and to aid researchers wishing to study BB function in zebrafish, we have extracted BB- and centrosome-specific genes from the SCGS list and cross-referenced against genes with functional data in zebrafish (Table 1). Out of the 60 BB-/centrosome-specific proteins extracted from the SCGS list, 29 showed zebrafish functional follow-up studies, with the majority limited to knockdown as opposed to knockout approaches of gene silencing. It is clear from our table that BB researchers are just beginning to realize the power of zebrafish to study vertebrate function of BB genes. With advanced genome editing techniques now accessible in zebrafish, we expect some insightful BB zebrafish papers to follow.

**Table 1:** Current zebrafish functional analysis, with zebrafish-specific references, of genes identified through the SCGS list to be BB/centrosome affiliated

Gene	RNA refseq/genbank	Associated disease	Genetic manipulation	Phenotype	Refs.
*Ahi1*	NM_001077561.1	JSRD	MO	CE, V, E, K, Ct, Hc, LRP, C−	[68]
*Cep131*	XM_009306856.1	BBS, T2D	MO	CE, E, LRP, BB+ , CLO	[15]
*Bbs10*	NM_001089463.1	BBS, T2D	MO	CE	[69, 70]
*Bbs12*	XM_002667206.3	ADPKD, NPHP, AS, OFDS, MKS, JS	MO	CE, LRP	[71]
*Bbs5*	NM_200299.1	BBS	MO	V, K, CLO	[72]
*C2orf71*	BI878361.1	RP	MO	V	[73]
*Cep83*	XM_009300427.1	Unknown	MO	E, CLO	[74]
*Cep164*	EB913016.1	NPHP	MO	CE, Mc	[75]
*Cep290*	NM_001168267.1	LCA, BBS, MKS, NPHP	MO	CE, V, K, Hc	[76, 77]
*Cep41*	NM_001002194.1	JSRD	MO	V, E, Hc, LRP, CM	[78]
*Disc1*	NM_001142263.1	SCZD	MO	V, Ct, Mc	[79]
*Kif7*	NM_001014816.1	ACLS, JS, HYLS	MO, ZFN Mt	Hh	[80, 81]
*Mks1*	NM_001077373.2	BBS, MKS	MO	CE	[82]
*Nek2*	NM_201050.1	RP	MO	V	[83]
*Nin*	XM_009307506.1	SS	MO	V, E, Mc	[84]
*Ninl*	NM_001281798.1	USH2A	MO	V, K, BB+	[85]
*Odf2a*	XM_001332528.6	MC	MO	V, Mc	[86]
*Poc1b*	NM_200118.1	CORD	MO	V, K, Ct, LRP, CLO	[53]
*Rab11a*	NM_001007359.1	Unknown	MO	LRP	[87]
*Rp2*	NM_213446.1	RP	MO	V, Hc, Mc	[88]
*Rpgrip1* *l*	NM_001246660.2	JS, MKS, COACH	MO	CE, V, Hc, LRP, BB+, CLO	[89, 90]
*Sass6*	NM_213438.1	MC	MO, ENU Mt	MD	[48, 91]
*Sdccag8*	XM_005156579.2	BBS, SLS	MO	CE, K, Hc	[92]
*Snx10a*	NM_001139462.1	O	MO	LRP, C−	[93]
*Stil*	NM_173244.2	MC	MO, ENU Mt, RVI Mt.	V, Mc	[94, 95]
*Toporsa*	NM_001305555.1	RD	MO	CE, V, Hc	[96]
*Ttk*	NM_175042.2	Unknown	ENU Mt	CE	[97]
*Yap1*	NM_001139480.1	Unknown	MO	CE, V, K, Hc, BB+, C−, CLO	[29]
*Gmnc*	XM_009291838.1	Unknown	MO, CRISPR Mt	K, C−, CLO	[32]

Note the addition of post-SCGS genes, *yap* and *gmnc*

*JSRD* joubert syndrome and related disorders, *BBS* bardet biedl syndrome, *T2D* type 2 diabetes*, ADPKD* autosomal dominant polycystic kidney disease*, NPHP* nephronophthisis*, AS* alström syndrome, *OFDS* orofaciodigital syndrome type 1*, MKS* meckels syndrome*, RP* retinitis pigmentosa*, LCA* leber’s congenital amaurosis*, MC* microcephaly*, USH2A* usher syndrome 2A*, COACH* cerebellar vermis oligophrenia ataxia coloboma hepatic fibrosis*, SCZD* schizophrenia*, SLS* senior-loken syndrome*, O* osteopetrosis*, CORD* cone-rod dystrophy*, RD* retinal degeneration*, ZFN* zinc finger nuclease, *ENU N*-ethyl-*N*-nitrosourea, *RVI* retroviral insertion, *Mt* mutant. Phenotype abbreviations: *CE* convergent extension defects, *V* visual impairment, *E* ear and otolith defects, *K* kidney defects including pronephric tubule dilation, *Ct* abnormal cartilage development, *Hc* hydrocephaly, *Mc* microcephaly, *LRP* left–right patterning defects, *BB*+ BBs observed intact, *C*− cilia absent, *CM* defective cilia motility, *CLO* cilia length and organization affected, *Hh* hedgehog signaling abrogated, *MD* mitotic division disrupted

### Notable basal body findings

Forward genetic mutagenic screens performed in the 1990’s, spearheaded zebrafish to the forefront of vertebrate functional genetic research. Teams from Boston (USA) and Tubingen (Germany), lead by Wolfgang Driever and Christiane Nusslein-Volhard, recovered hundreds of *N*-ethyl-*N*-nitrosourea (ENU) directed mutations that caused gross morphological abnormalities in zebrafish development [39, 40]. At the time of screening, the significance of cilia in human disease had not been determined. Mutants identified through screening processes were grouped together based on common phenotypic features. One group of mutants showed phenotypic similarities to the ift88 mouse, a gene that had been shown in *chlamydomonas* to be required for ciliogenesis. Now considered the archetypal zebrafish ciliopathy phenotype, mutant lines display randomized heart looping and laterality defects, curved body axis, hydrocephalus, pronephric and glomerular cysts, and defective eye development [41]. Several of these mutations have been mapped to key components in ciliary processes. Notably affecting components of the IFT system. For example, the zebrafish mutants oval (ift88), fleer (ift70), and elipsa (traf3ip1), display loss of ciliary assembly [42–45]. However, these mutants have intact BBs, suggesting that the BB alone is not sufficient for ciliogenesis.

Early zebrafish ENU screens appeared to recover mainly ciliary/axonemal gene mutations, rather than those specific to basal body construction or function, although a number of mutants still remain unmapped. More BB/centriolar relevant mutants have been discovered through genetic screens for maternal-effect mutations [46, 47]. These experiments set out to understand the maternal factors required for early embryonic development and in doing so, identify genes involved in the early cell cycle events that occur before zygotic genes switch on. As previously mentioned, cilia do not form in zebrafish until late gastrulation (approximately 9-h post-fertilization (hpf)), suggesting that any centriolar mutations will be more akin to the centrosome [26]. Interestingly, one of the mutants recovered, a missense mutant (Asn414Lys) known as cellular atoll (cea), encodes the centriolar component Sass6 [48]. Genotypically homozygote cea individuals develop to adults and look phenotypically identical to wildtype, however females produce clutches of eggs that due to defects in centrosomal duplication arrest during early cell division. Thus, Sass6 is a maternal effect gene required for pre-gastrulation centrosomal duplication in zebrafish. However, the single amino acid change in cea appears not to affect BB function, homozygotes are viable and develop to adulthood. In other organisms, Sas-6 is localized to the centriolar cartwheel and has been speculated to form the cartwheel hub where loss leads to aberrant triplet microtubule numbers [19, 20, 49]. Thus, Sas-6 localizes to the cartwheel hub and is essential for centriole symmetry. Indeed, x-ray crystallography of zebrafish Sas-6 N-terminal has revealed that it assembles itself in vitro into constructs reminiscent of cartwheel hubs [21]. Further work on zebrafish, with the development of conditional mutations, will be critical in understanding the role of vertebrate Sas-6 in BB function.

Zebrafish forward genetic screens have been instrumental in understanding gene function, however mutations for genes of interest are not always recovered. A popular choice, although recently called under scrutiny, is the use of antisense morpholino oligonucleotide technology (MO) to block gene-specific translation [50, 51]. MOs are cheap to synthesize, easy to administer and fast to generate preliminary data. Furthermore, since MOs provide gene knockdown rather than knockout their use maybe more favorable for understanding gene function required for very early stages of development, such as cellular division, when early lethality otherwise masks ENU mutation recovery. Several zebrafish studies have utilized MOs to study basal body protein function in vertebrates. A notable case is that of Poc1, a core centriolar WD40 domain protein identified in both *Chlamydomonas* and *Tetrahymena* centriolar proteomic screens [34, 37, 52]. Interrogation of Poc1b function in *Tetrahymena* revealed a structural role in BB stability [53]. Knockdown of the zebrafish orthologue Poc1b using MOs results in phenotypic similarities to cilia deficient mutants, including visual impairment. Cilia motility and length is hindered in Poc1b morphant zebrafish embryos [53–55]. Recently, mutations in POC1B have been identified in patients displaying ciliopathy features [54, 56, 57]. Together, these data show the power of multidisciplinary research that can ultimately lead to the identification of novel disease causing genes.

### Strengths and future of basal body research in zebrafish

The many advantages of using zebrafish as a model organism has firmly established this small tropical aquarium fish as a popular laboratory aid. Their rapid development, production of large numbers of eggs, optical transparency and excellent value for money are very appealing to vertebrate researchers. Additionally, BBs can be easily visualized alongside cilia in multiple zebrafish tissue by using primary antibodies for γ-Tubulin (BB—GTU88 Sigma) and acetylated α-Tubulin (Cilia—T6793 Sigma) in conjunction with isotype-specific secondary antibodies (Fig. 1e–g) [58]. For many years, a major drawback when modeling gene function in zebrafish was the difficulty in performing targeted mutagenesis. As such, zebrafish researchers have relied on MOs to knockdown gene-specific translation, a relatively quick and inexpensive technique [59]. However, problems associated with MO off-target defects have meant that an arduous list of controls need implementing in order to validate MO induced phenotypic changes [60, 61]. In the last few years, techniques to provide targeted mutagenesis in zebrafish have rapidly evolved thanks to the use of genome editing tools such as TALENS and CRISPR [62, 63]. Their development has highlighted some of the inaccuracies in the literature that have spread through MO use, where as many as 80 % of MOs may actually fail to recapitulate bona fide mutations in genes of interest [50]. CRISPR and TALENS take advantage of the imperfect endogenous repair mechanism, non-homologous end joining, which initiates after targeted double stranded DNA breaks are induced by certain endonucleases (reviewed in: [64, 65]). The development of tissue-specific promoter driven endonuclease expression has enabled researchers to create conditional mutants [66]. Minimal knowledge of molecular biology is required to generate the reagents required to direct the CRISPR Cas9 endonuclease to a favorable region of the genome, making this available to most laboratories and favorable over TALENS. In addition, there are comprehensive published protocols to perform, validate, and maintain CRISPR-induced mutagenic lines [66, 67]. Therefore, generating CRISPR directed mutant zebrafish lines is fast becoming an established method in zebrafish laboratories. Yet, there is little published work on BB-specific mutant zebrafish lines. Both global and conditional CRISPR techniques will provide BB researchers with invaluable tools to study candidate gene function, especially when considering the ubiquitous nature of BB gene expression. There is huge scope for utilizing zebrafish in BB research and it will be exciting to see how the systematic mutagenesis of the BB proteome will identify novel roles both at the structural and functional level.

### Ethics statement

*Animal* maintenance, husbandry, and procedures are defined and controlled by the Animals (Scientific Procedures) Act 1986. All animal experimentation has been carried out under licenses granted by the Home Secretary (PPL No. 70/7892) in compliance with Biological Services Management Group and the Biological Services Ethical Committee, SGUL, London, UK.

## Abbreviations

BB: basal bodies
WGD: whole genome duplication
IFT: intraflagellar transport
TEM: transmission electron microscopy
MCC: multiciliated cells
PCM: pericentriolar material
SGSC: Syscilia’s Gold Standard
ENU: *N*-ethyl-*N*-nitrosourea
Hpf: hours post-fertilization
MO: antisense morpholino oligonucleotide
TALENs: transcription activator-like effector nucleases
CRISPR: clustered, regularly interspaced, short palindromic repeat
JSRD: joubert syndrome and related disorders
BBS: bardet biedl syndrome
T2D: type 2 diabetes
ADPKD: autosomal dominant polycystic kidney disease
NPHP: nephronophthisis
AS: Alström Syndrome
OFDS: Orofaciodigital syndrome type 1
MKS: Meckels syndrome
RP: Retinitis pigmentosa
LCA: Leber’s congenital amaurosis
MC: microcephaly
USH2A: Usher syndrome 2a
COACH: cerebellar vermis oligophrenia ataxia coloboma hepatic fibrosis
SCZD: schizophrenia
SLS: Senior-Loken syndrome
O: osteopetrosis
CORD: cone-rod dystrophy
RD: retinal degeneration
